# Prof. Hobday’s Results in Median Nenrectomy

**Published:** 1897-02

**Authors:** 


THIRTY-EIGHT cases of median neurectomy are reported by Professor Hobday, with thirty-one favorable results, two unfavorable, two still under treatment, and three untraced. The lesions were in nearly all instances located between the knee and the fetlock.—*Veterinary Record.*

